# Chloroplast genome and phylogenetic analysis of *Grewia biloba*

**DOI:** 10.1080/23802359.2021.1932626

**Published:** 2021-05-27

**Authors:** Dongya Xu, Meng Liu, Haozhan Ren, Bin Zhang, Zongcai Liu, Hongwei Wang

**Affiliations:** a College of Life Science and Agricultural Engineering, Nanyang Normal University, Nanyang, China; b College of Plant Protection, Henan Agricultural University, Zhengzhou, China

**Keywords:** *Grewia biloba*, chloroplast genome, phylogenetic analysis

## Abstract

*Grewia biloba* is a potential medicinal and ornamental resource, and this study is the first to assemble the chloroplast genome of this species using high-throughput sequencing data. The chloroplast genome of *G. biloba* has a typical composition for higher plant chloroplasts, containing one large single-copy region of 86,978 bp and one small single-copy region of 20,140 bp, which are separated by a pair of inverted-repeat regions of 25,473 bp. The chloroplast genome of this species encodes 130 genes, including 84 protein-coding genes, 38 tRNA genes and 8 rRNA genes. A maximum-likelihood phylogenetic tree was constructed based on sequence information of the chloroplast genomes. The chloroplast genome of *G. biloba* will provide valuable genetic information for evolutionary research and utilization of this species.

*Grewia biloba* G. Don is a deciduous shrub or small tree of the genus *Grewia* in the family Malvaceae. This species is primarily distributed in southern China, with a small number observed in northern China. In some Asian countries and regions, several plants of this genus are used as folk medicinal herbs and are occasionally cultivated in gardens as ornamental plants (Yang and Gao 2013). A recent pharmacological study has shown that ethanol extracts of *G. biloba* root bark and solvent extracts with different polarities had antitumor effects on cervical cancer growth in mice (Liu et al. 2008). An *n*-butanol extract from *G. biloba* branches showed antibacterial, antimalarial and anti-inflammatory effects (Yang and Gao 2013). In addition, an ethyl acetate fraction of *G. biloba* showed analgesic effects, which might be related to the inhibition of prostaglandin E2 and malondialdehyde, as well as the promotion of nitric oxide release (Yang et al. 2014). In this study, we assembled the chloroplast (cp) genome of *G. biloba* using high-throughput sequencing data and constructed a phylogenetic tree based on cp genome information of related species, which may help establish a foundation for evolutionary research, development and utilization of this species.

Fresh leaf samples of *G. biloba* were collected on the campus of Henan Agricultural University (34°47′7″N, 113°39′35″E), Zhengzhou city, China. The specimens (MALGR20190815) were preserved in the herbarium of Henan Agricultural University. Total genomic DNA was extracted using a modified cetyltrimethylammonium bromide method (Fang et al. 2009). The purified DNA was subsequently fragmented and used to construct short-insert libraries according to the manufacturer’s instructions (Illumina) and then sequenced on an Illumina HiSeq 4000 system (Borgstrom et al. 2011). After the raw reads were filtered, the clean reads were assembled into circular genomes using GetOrganelle (Jin et al. 2018). Annotations and adjustments of genes were conducted manually using Geneious Prime (Kearse et al. 2012). The complete cp genome of *G. biloba* was submitted to GenBank (accession number: MW699774).

The cp genome of *G. biloba* is 158,064 bp in length and has a typical structure of a higher plant cp genome; that is, it contains one large single-copy region and one small single-copy region, which are separated by a pair of inverted-repeat regions. The sizes of the above three regions are 86,978, 20,140 and 25,473 bp, respectively. The cp genome of this species encodes 130 genes, including 84 protein-coding genes, 38 tRNA genes and 8 rRNA genes. The GC content of the whole genome is 37.4%, which is similar to that of other species in this family (Alzahrani 2021; Wang et al. 2021).

The assembled cp genome of *G. biloba* was aligned with the cp genomes of 24 other Malvaceae species, which were downloaded from the NCBI database using the MAFFT method in Geneious Prime (Kearse et al. 2012). A maximum-likelihood (ML) phylogenetic tree was subsequently constructed using RAxML-HPC2 on XSEDE v8.2.12 on the CIPRES cluster (Miller et al. 2010). In the constructed phylogenetic tree, *Colona floribunda* grouped with another species of the genus *Grewia*, *G. chungii*, and formed a sister clade with *G. biloba* (Figure 1). The cp genome of *G. biloba* may provide valuable genetic information for the study of phylogenetic relationships.

**Figure 1.**
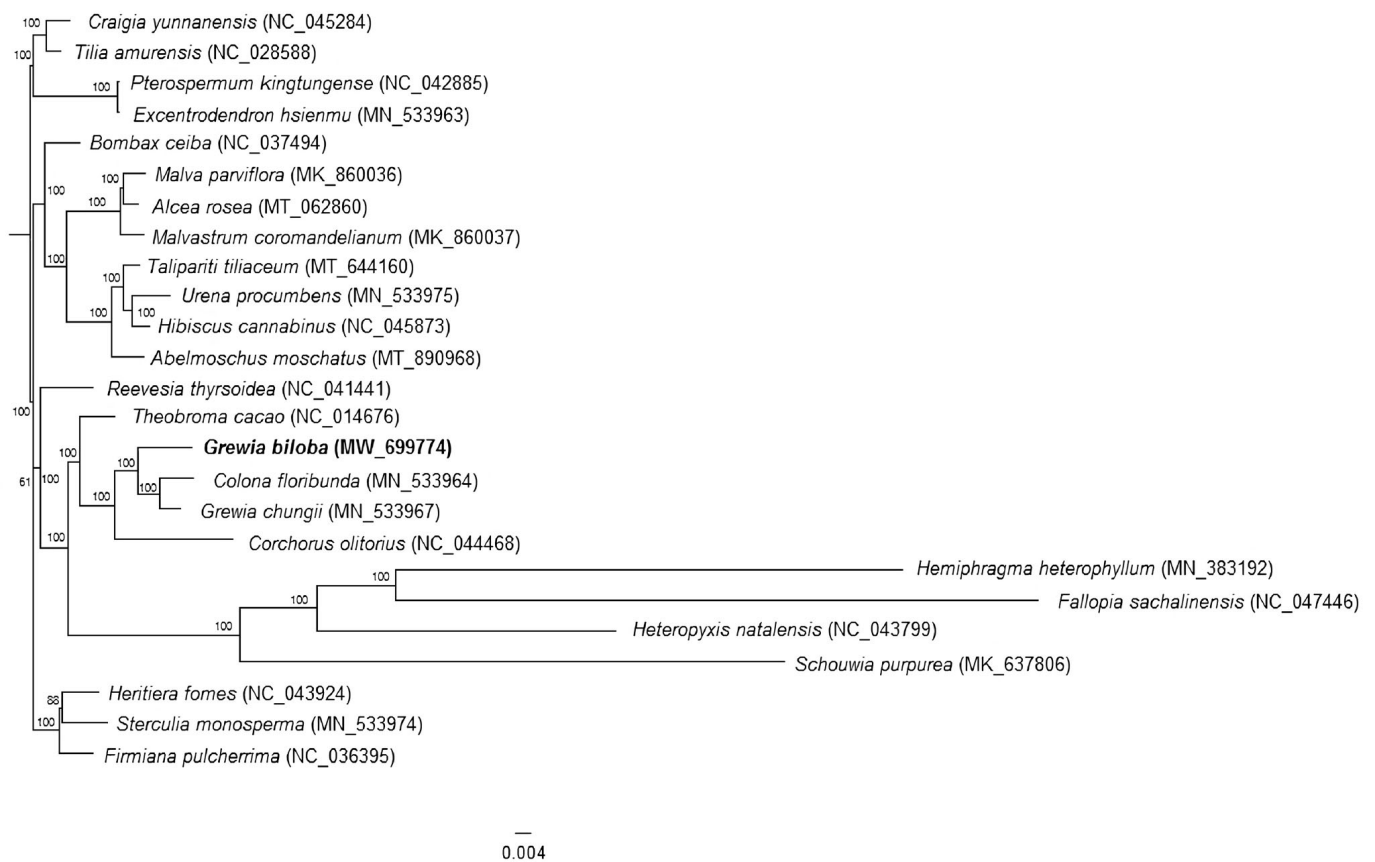
ML phylogenetic tree of 25 species of Malvaceae based on cp genomic information. Bootstrap support values are shown next to nodes. The new complete cp genome obtained in this study is shown in bold.

## Data Availability

The genome sequence data that support the findings of this study are openly available in NCBI GenBank (https://www.ncbi.nlm.nih.gov/nuccore/MW699774). The associated BioProject, SRA, and Bio-Sample numbers are PRJNA707319, SRR13883638, and SAMN18200682, respectively.
